# Ultrasensitive fluorescent detection of pesticides in real sample by using green carbon dots

**DOI:** 10.1371/journal.pone.0230646

**Published:** 2020-03-24

**Authors:** Fatemeh Ashrafi Tafreshi, Zahra Fatahi, Seyedeh Fatemeh Ghasemi, Amirali Taherian, Neda Esfandiari

**Affiliations:** 1 Faculty of Life Sciences and Biotechnology, Shahid Beheshti University, Tehran, Iran; 2 Protein Research Center, Shahid Beheshti University, Tehran, Iran; Consiglio Nazionale delle Ricerche, ITALY

## Abstract

Pesticides, widely used in modern agriculture, could potentially cause environmental pollution and affect human lives. Hence, the development of a highly sensitive sensing element to detect pesticide residues is crucial for food safety and ecosystem protection. Optical methods based on fluorescence properties provide an ideal approach for screening and quantification of these compounds in different medias including water, plant, and nutritional products. The development of fluorescence emitting carbon dot-based sensors for monitoring pesticides has attracted great attention in recent years. In comparison to other fluorophores, carbon dots have more promising optical features, higher quantum yields and better biocompatibility. This article aims to present a novel fluorescent sensing method of diazinon, glyphosate, and amicarbazone using plant-based carbon dots. A comprehensive characterization of carbon dots obtained from cauliflower was performed by methods including UV-visible, FTIR spectroscopy, fluorometry, AFM, DLS, and zeta sizer. Following this step, carbon dots were used to detect pesticides. The fluorescence quenching property of carbon dots has been utilized to identify detection limit of 0.25, 0.5, and 2 ng ml^-1^ for diazinon, amicarbazone, and glyphosate, respectively. Also, real sample study revealed that the detection of pesticides accompanied by our developed nano-sensor is repeatable and accurate. According to carbon dots specificity determination, the prepared nano sensor does not have the potential to identify “bromacil” and “dialen super” pesticides but the other three mentioned pesticides are detectable. The results confirm that synthesized green carbon dots are well qualified for application in food safety and environmental monitoring.

## Introduction

Pesticides are generally used in agriculture to prevent, control and eradicate weeds and pest from interfering with the crops production chain. These involve insecticides, herbicides, and various pest control substances which are used to meet the demands of an ever-increasing population[1]. Since the development of pesticides, a significant amount of them have been consumed each year. Accordingly, there are great amount of pesticides releasing into the environment and ecosystem. Furthermore, the common detection strategies are not capable to detect low quantities of pesticides and the remaining residues contaminate the surrounding environment [2]. Moreover, the pesticide residues could cause serious health problems for living organisms even in low concentrations [3]. The discussed issues express a firm requisition to monitor such chemicals in the environment and consumable products.

Diazinon (phosphorothioic acid O, O-Diethyl O-[4-methyl-6-(propan-2-yl) pyrimidin-2-yl]) is known as an organophosphate (OPs) pesticide with a broad spectrum of insecticide activity against various pests of fruits, vegetables, field crops, grasslands, and ornamental plants. Unfortunately, such pesticide remains stable for up to six months in water and causes irreversible biochemical changes. It could also be ubiquitous in the environment and presents highly toxic effects on the immune and neurological systems, from wildlife organisms to amphibians [4–6]. In spite of organophosphates toxicity to human beings and their widespread concern, they are still used as pesticides [7]. It is expected that extended use of OPs would possibly influence hygiene and mankind environment [8]. Regrettably, OPs have the potential to inhibit acetylcholinesterase (AChE) crucial enzyme and affect its longevity. This leads to accumulation of acetylcholine in cholinergic synapse which results in threatening human life [9,10].

In the matter of controlling weeds, amicarbazone (4-amino-N-(1, 1-dimethyl ethyl)-4, 5-dihydro-3-(1-methyl ethyl)-5-oxo-1H-1, 2, 4-triazole-1-carboxamide) is noteworthy to be mentioned. Amicarbazone originates from triazolinone herbicide with significant applications in weed dominance within the corn and sugar fields. It acts as a photosynthesis inhibitor which leads to chlorosis, stunted growth, tissue necrosis, and eventually eradication of weeds [11]. However, it has been proved that unprofessional use of these chemicals could lead to human and animal affection which could cause serious health problems [12].

Moreover, Glyphosate (N-(phosphonomethyl) glycine) as one of the largely used pesticides in the world, has the value to be discussed. Glyphosate has the potential to inhibit 5-enolpyruvylshikimate-3-phosphate synthase (EPSPS), in the shikimate pathway. The inhibition of such enzyme leads to downgrade the pathway to produce aromatic amino acids and secondary metabolites such as lignin [13]. However, according to records that reported the resistance of some weeds due to the increase in glyphosate usage, it has made a global concern over the use of such pesticide [14].

There are several laboratory-based methods being carried out for the detection of the pesticides such as high-performance liquid chromatography (HPLC), gas chromatography (GC), capillary electrophoresis (CE), and mass spectrometry [15]. Although the mentioned methods are sensitive and display accurate results, significant number of such methods are laboratory-based, time consuming, and inapplicable for point-of-care detection [16]. Therefore, a simple, sensitive, and cost-effective method **w**as designed for detecting pesticides, which is based on optical property. Optical based analytical probes provide a good condition of analysis since they often involve low cost reagents, simple instruments, short response time, and effortless to perform. A wide range of fluorescent-based sensors have been designed including organic dyes, quantum dots, metal-organic frameworks, fluorescent proteins, etc. Carbon dots (CDs) which have been discovered in 2004 during separation and purification process of single walled carbon nanotubes, have considered as promising in the last decade due to their outstanding optical properties, biocompatibility, negligible toxicity, great solubility, and facile synthesis. CDs are typically semi-spherical including amorphous to nanocrystalline, naturally consist of oxygen-nitrogen-based groups, and post-modified functional groups which cause easy interaction and binding to target molecules. CDs could be derived either from natural or non-toxic precursors which are inexpensive and appropriate for large scale applications [17]. The convenience of synthesis and unique properties of CDs have led to enormous attention for employing different kinds of precursors and synthesis methods, which were mainly classified as top-down and bottom-up approaches [18–21]. The “top-down” approach includes techniques such as arc-discharge [22], laser ablation [23], and electrochemical exfoliation [24]. As the “bottom-up” method contains microwave treatment [25], sonochemical treatment [26], thermal decomposition [27], and thermal treatment [28]. Among the above-mentioned methods, hydrothermal treatment is one of the most prevalent bottom-up method being used to synthesize CDs form peels or juices of fruits, known as green carbon sources. Hydrothermal carbonization provides simple one-step experimental setup through high temperature and pressure which avoids the need of highly toxic chemicals. Furthermore, a cost effective and sustainable source of raw and biowaste materials should be utilized for the synthesize of CDs [29]. Additionally, efforts to synthesize fluorescence emitting CDs have been made to use in sensors [30]. Carbon dots-based sensors have enormously been used for metallic ions detection such as Hg^**2+**^ [31], Cu^2+^ [32], K^+^ [33], Ag^+^ [34], and Fe^3+^ [35]. Therefore, sensors could be used for the detection of a vast number of biological agents and materials. One of CDs green precursor is cauliflower (*Brassica Oleracea*) from the *Brassicaceae* family (syn. *Cruciferae*) which contains many organic compounds such as gallic, pyrogallol, catechin, protocatechuic, chlorogenic acid, rosemary acid, rutin, caffeic acid, vanillic acid, quercetin, naringenin, syringic acid, coumaric acid, cinnamic acid, and kaempferol [36]. These particular bioactive compounds could bring the right and crucial elements to synthesize carbon dots.

**Fig 1:**
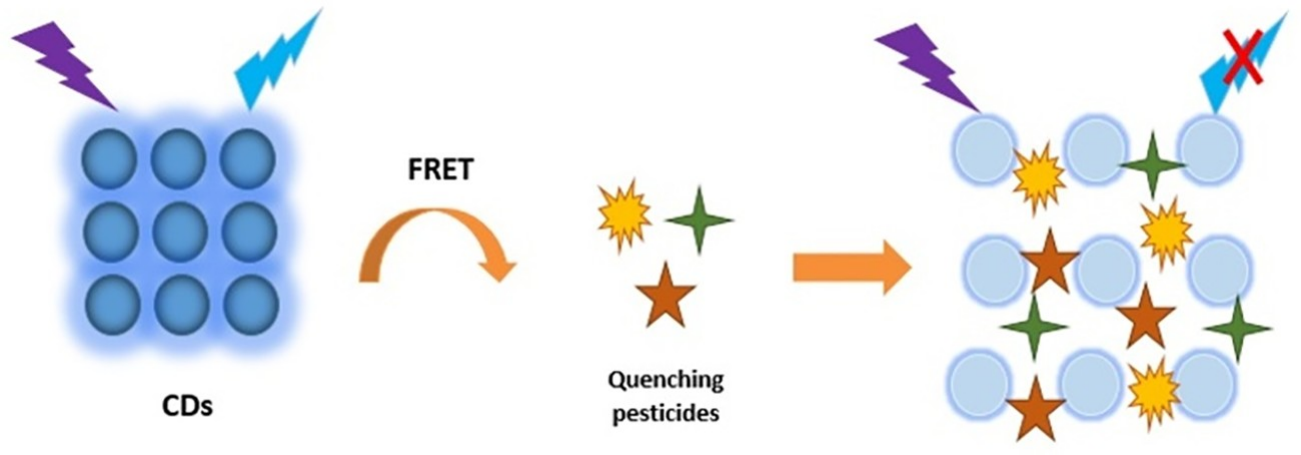
CDs fluorescence quenching by pesticides based on FRET mechanism. CDs as donors and three different pesticides as acceptors.

Various CDs-based sensors have been designed in the area of food safety for pesticides detection [37]. Important analytical strategies for pesticides detection include the inhibition of the enzymic activity of the pesticides and other method follows the principle of the inner filter effect (IFE) and florescence resonance energy transfer (FRET). In the first method, metabolites from the enzymic activity quench the CDs fluorescence emission. Moreover, the presence of pesticides in solution inhibit the enzymic activity and the quenchers which leads to recurrence in CDs fluorescence emitting [38,39]. FRET (Fig 1) and IFE require two determinant factors, first, the overlap of the absorption, and emission spectrum, and second, the distance between CDs as a donor and quencher or acceptor. The difference is that FRET usually occurs at less than 10 nm distance, while IFE mechanism turns up at more than 20 nm distance. However, they are both more sensitive and selective than enzyme catalysis [37,40,41].

In this inquiry, CDs were prepared by using a one-step hydrothermal treatment of pesticides free cauliflower. The prepared CDs were used for the detection of diazinon, glyphosate, and amicarbazone pesticides. This method is highly reproducible, sensitive, cost-effective, target for real samples, and easy to perform. Moreover, to evaluate the accuracy of this sensing probe, the test was carried out in cherry tomato as a real sample. Thus, by measuring the fluorescent intensity of green CDs, a turn-off fluorescent based sensor for the detection of diazinon, glyphosate, and amicarbazone was established.

## Materials and methods

Diazinon, glyphosate, amicarbazone, bromacil, and dialen super were donated from Dr. Pourrahim. Pesticide free cauliflower as a precursor has been bought from an organic local market (Tehran, Iran). Deionized (DI) water was purified using the Milli-Q System (Iran).

### Synthesis of carbon dots (CDs)

**Fig 2:**
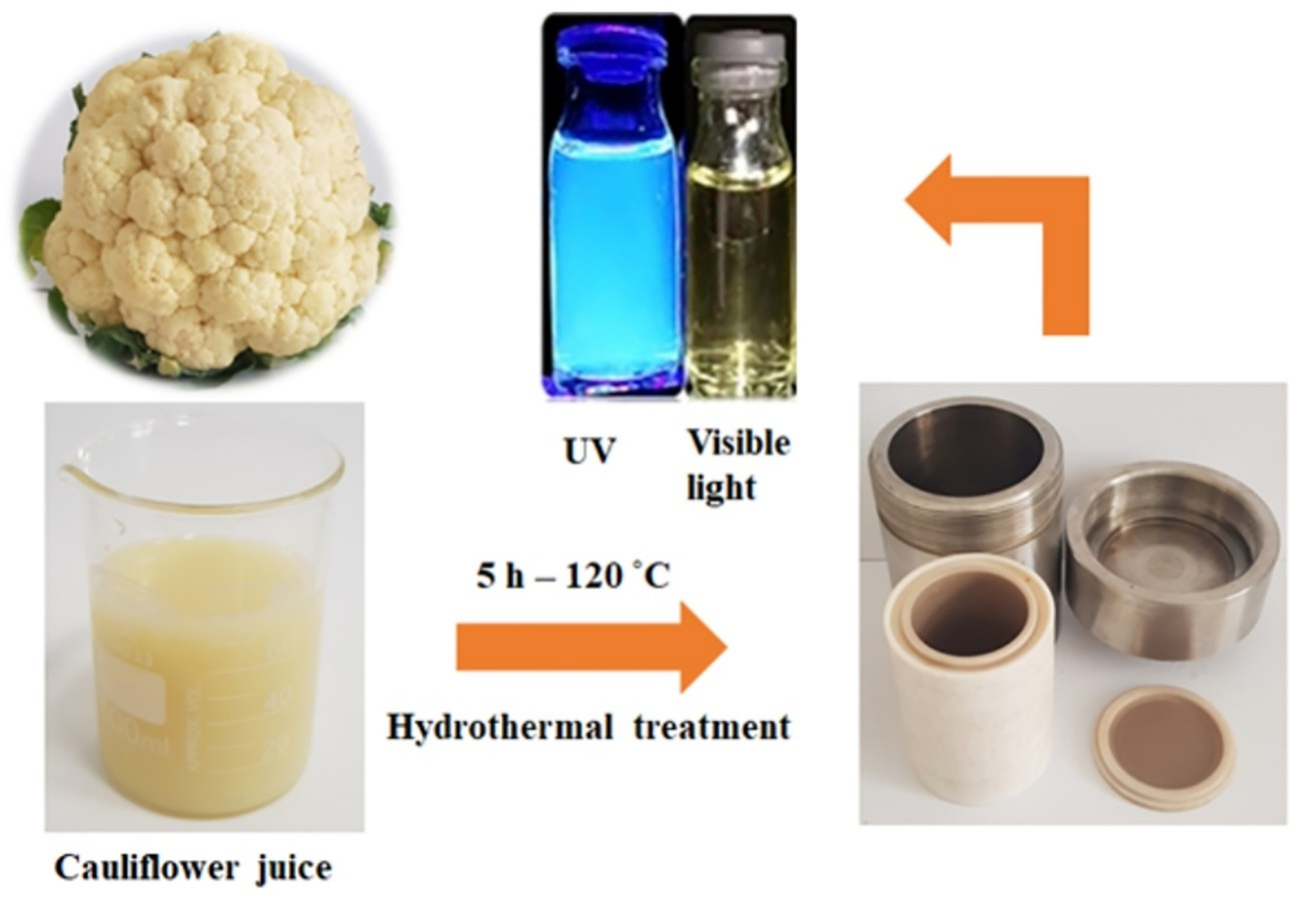
Schematic illustration of CDs preparation from hydrothermal treatment of cauliflower. The cauliflower plant was transferred to Teflon-lined autoclave after the juice was extracted. Then, the extracted juice was heated at 120°C for 5 h. The prepared CDs emitted bright blue illumination under UV light irradiation. After the heating process, the solution turned into brown color which is an evidence of carbonization.

The fluorescence emitting CDs were synthesized through hydrothermal carbonization approach using pesticide free cauliflower juice as a carbon source. In a typical synthesis, as shown in Fig 2, the cauliflower plant was washed off with distilled water and then the juice was extracted with a juicer machine. Following the first step, the mixture was filtered to remove large, biomass-based aggregates. Then, 60 ml of filtrated juice was moved to a 100 ml Teflon-lined stainless-steel autoclave and was heated at constant temperature of 120°C for 5 hours in the oven and then allowed to cool down. The obtained dark brown solution was centrifuged at 9000 rpm for 15 minutes and filtered with a 0.22 μm membrane to remove insoluble particles. Finally, the products were dried by a freeze dryer and were achieved to the desired concentration (1mg ml^-1^). The final solution was stored at 4 °C for further use.

### Characterization techniques for CDs

Chemical compounds, size, topology, and structure are the most important factors that determine the characteristics of CDs.

To evaluate the synthesized solution’s fluorescent intensity, first, the solution was observed in a UV cabin. Afterward, UV-Vis absorption spectra were recorded with PerkinElmer Lambda 2 UV-visible spectrophotometer. CDs prepared by typical synthesis methods usually exhibit strong ultraviolet (UV) absorption. However, the wavelength of UV absorption peaks depends on different synthetic approach [42]. The fluorescence emission spectrum was assessed with PerkinElmer LS 45 fluorescence spectrophotometer. CDs’ solution was diluted 2 folds to clarify optical property of nanoparticles. As size is the main factor to consider a particle in nano scale, particle size was measured with dynamic light scattering (DLS, Nanophox, Sympatec GmbH, Germany) and also surface electric charge was justified with zeta potential analyzer (ZEN 3600, Malvern Instruments CO., USA). DLS is a common technique to determine the size distribution of small particles by measuring the changes in the intensity of light that scattered from a suspension. Zeta potential can be determined through the particle’s movement due to the interaction between the charged particle and applied field[43,44]. Furthermore, atomic force microscopy (AFM) was used to determine morphology properties (Nanosurf, Switzerland). The sample was prepared by pipetting a few microliters of the CDs’ solution on the Mica wafer after sonication. Small piezoelectric ceramics probe is scanned across the specimen in the X, Y, and Z directions to achieve information about the sample’s surface[43]. Studies on chemical composition and the surface state of synthesized CDs were also observed with Thermo Nicolet Fourier transform infrared spectroscopy (FTIR) at wavenumbers ranging from 500 cm^−t^ to 4000 cm^−t^. CDs are generally composed of carbon, oxygen and hydrogen. As CDs are obtained by partial oxidation of carbon precursor, the surface of CDs is rich in hydroxyl, epoxy/ether, carboxyl or carboxylic acid groups. FTIR is a practical instrument for analysis of these groups [42].

### Quantum yield calculation

Quantum yield (QY) is a quantity that demonstrates the quality of the synthesized structure. It could draw a comprehendible connection between emitted photons from a fluorescence material and their absorbed photons. Also, the refractive index for synthesized CDs and standard material was about 1.33.

Quantum yield is computed with equation below:

$${QY}_{x}={QY}_{st}\times \frac{A_{st}\times I_{x}\times {ƞ}_{x}^{2}}{A_{x}\times I_{st}\times {ƞ}_{st}^{2}}$$ (1)

Formula (1), “*QY*” indicates quantum yield, “A” stands for absorption or optical density quantity, “I” states emission value in stimulator wavelength and “ƞ” indicates wavelength refractive index value. Moreover, “st” and “x” index show the relative value of standard material and carbon dots, respectively[45,46]. According to previous studies, high quantum yield could also be an attributing factor in better selectivity and lower detection limit of carbon dots- based sensors [47].

### Detection of pesticides

The synthesized CDs are used as instrument for the detection of pesticides. Consequently, in this study, diazinon, glyphosate, and amicarbazone were selected as a model to evaluate CDs detection efficacy. CDs solution, owning to their highest fluorescent emission and ultrapure water were considered as positive and negative control respectively. Therefore, CDs solutions (1μl and 1mg ml^-1^) were added to different concentrations of mentioned pesticides (0.25, 0.5, 2, 8, 40, 200, 1000, and 5000 ng ml^-1^). To evaluate quenching efficacy, the fluorescence intensity of CDs was evaluated in presence of amicarbazone, glyphosate, diazinon in mentioned concentration. Three pesticides were also presented together in the same sample with the same concentration of each pesticides individually. The quenching effect of the three pesticides have also been assessed.

To investigate the specificity of the prepared sensor, dialen super and bromacil herbicides which control weed growth [48,49] were examined at different concentrations of 0.25, 0.5, 2, 8, 40, 200, 1000, and 5000 ng ml^-1^. Moreover, the fluorescence intensity of CDs in the presence of dialen super and bromacil were observed with a UV light and fluorescence spectrophotometer. Eventually the results were compared together.

### Pesticides detection in real sample

To further confirmation of CDs detection efficiency, pesticide-free cherry tomatoes were selected as an example of real sample to investigate the capability of this sensor in pesticides detection in real sample [50]. Another contributing factor in selecting such sample is that all tomatoes were picked from the same pesticide free shrub which can provide the equality of the properties among real samples. Cherry tomatoes were then exposed to the prepared solutions containing CDs (1μl, 1mg ml^-1^) and different concentrations (0.25, 0.5, 2, 8, 40, 200, 1000, and 5000 ng ml^-1^) of pesticides. Tomatoes that submerged in ultrapure water and CDs in a pesticide-free solution were considered as negative and positive control in respect. The fluorescence intensity of CDs in tomato was evaluated once per day for 5 days. A significant penetration of CDs into cherry tomatoes were observed at day 5th. According to that, a 5-day period was selected to clearly determine the penetration of CDs into cherry tomatoes. Then, cherry tomatoes juice was extracted and the fluorescence intensity of the solutions was observed with UV light and their fluorescence emission spectrums (excitation at 365 nm) were recorded, after 5 days. Also, this test was repeated three individual times.

### Statistical analysis

All experiments were treated and analyzed in triplicate. Mean and standard deviations were expressed and graphs were drawn with Origin Pro 2019 64-bit software. The significant of difference among these means were analyzed by ANOVA test (SPSS 16.0 software) and *p* ≤ 0.05 was considered as statistically significant.

## Result and discussion

### Synthesis and characterization of CDs

In this research, CDs were synthesized by carbonization of pesticide free cauliflower plant which contains sulfur compound that contribute in surface functional group of CDs with facile and cost-effective hydrothermal technique [51]. The hydrothermal method has become an approach to synthesize CDs from organic precursors. In this technique, organic materials undergo high levels of pressure and heat, aiming to form carbon dots. Possible processes to form CDs from organic resources could be procedures such as dehydration, fragmentation, condensation, aromatization, and carbonization [52].

**Fig 3:**
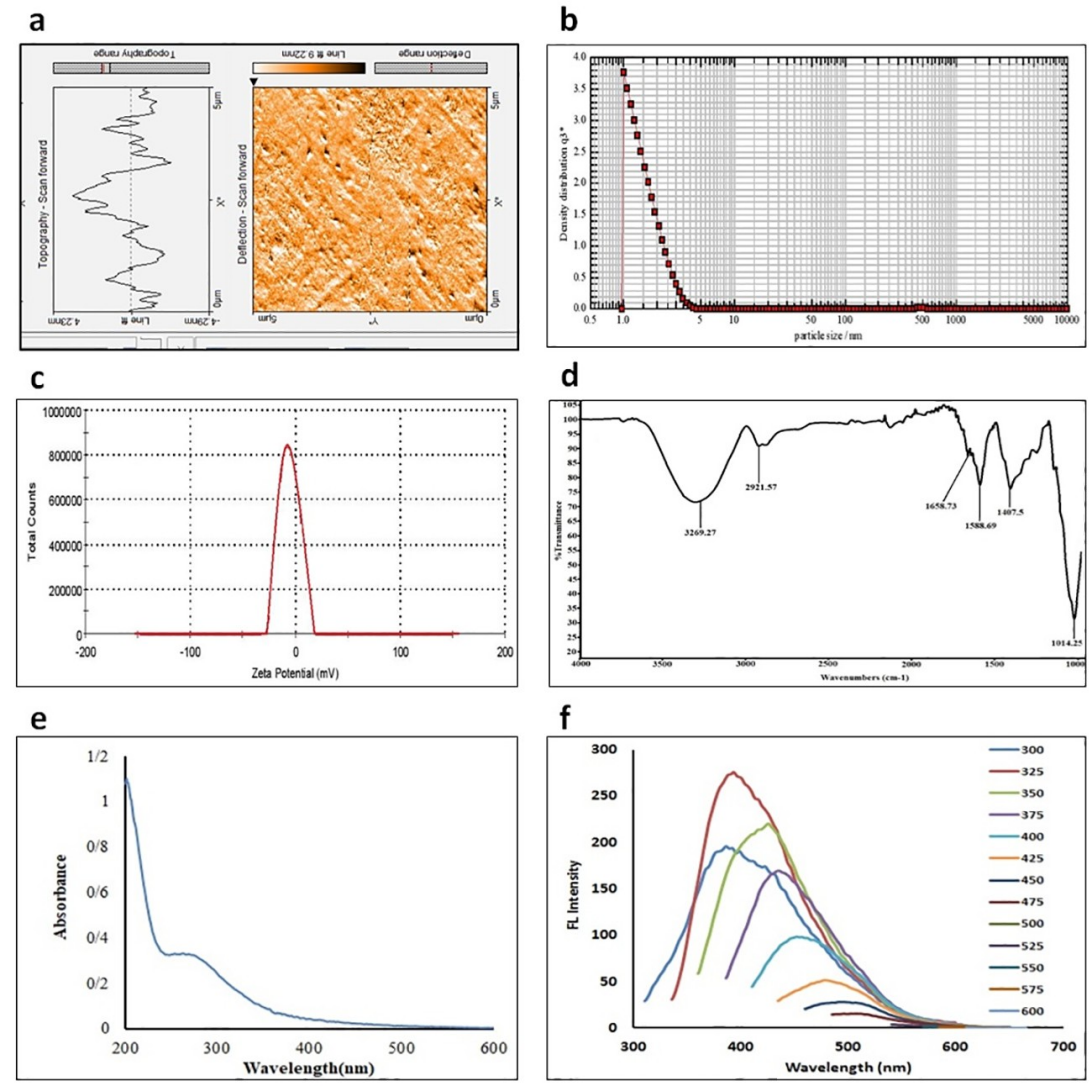
CDs characterizations analysis. (A) AFM analysis represents the morphology of nanoparticles and size of 4 nm. B) DLS analysis indicates CDs size of 1.54 nm (C) Zeta potential analysis of synthesized CDs which is used for particles’ zeta measurement approves -6.29 mv charge of prepared CDs (D) FT-IR analysis of CDs confirms the formation of different functional group including C = O, C-H, C-H_3_ O- H and N-H based on various absorption bands (E) UV-visible spectroscopy absorption of the aqueous dispersion carbon dots represents absorbance band at wavelength of 280 nm (F) excitation- dependent fluorescence spectra of CDs at different excitation wavelengths with the interval of 25 nm.

The prepared CDs were characterized by different techniques. The Morphology of CDs was investigated by AFM. As it shows in Fig 3A, height distribution was reported about 4nm and 3–5 layers of graphite have been detected. The achieved data has also been observed in previous studies[53,54]. According to other researches, size enlargement due to aggregation is not an unexpected fact. Herein, size increase of CDs has been observed in AFM measurements which has also been observed in other reports [55]. DLS analysis revealed that the diameter of CDs is 1.54 nm which gives an evidence to CDs monodispersed structure (Fig 3B). Additionally, the prepared nanoparticles were smaller in size in comparison to other studies[56,57]. According to Fig 3C, zeta potential evaluation presented negatively charged surface (-6.29 mV) of CDs. That implicates the presence of hydroxyl and carboxylate groups on the surface of the CDs as reported before [52,58–60]. In CDs FT-IR spectra (Fig 3D), 3269.27 cm^-1^ absorption band refers to O- H and N-H vibrations. Additionally, bands at 1588.69, 1407.5, and 1014.25 cm^-1^ are analogous to C-H, C-H_3_, and C-O bonds vibrations respectively. Eventually, C = O that accounts for CDs characteristics was detected at 1658.73 cm^-1^ which was compatible with other reports [61,62]. Consequently, the solubility of synthesized CDs was pledged by hydroxyl, epoxy, carbonyl, carboxylic acid, and amino-propyl groups. Also, the consistency of the results can be observed in other studies [58,63]. The UV- visible absorption and fluorescent emission spectra were performed to analyze the optical properties of obtained CDs. Referring to n–π* and π–π* of the C = O bonds of carboxyl group and conjugated C = C bonds conversion, an extensive absorbance band was observed at 280 nm as it was observed by UV-visible spectrum (Fig 3E). Moreover, other studies reported the exact results [64,65]. Fig 3F presents CDs emitted fluorescence spectra in the range of 300 to 600 nm and the excitation wavelengths with the interval of 25 nm. The optimum fluorescence intensity was observed at the excitation wavelength of 325nm. CDs also have presented a great emission peak at an excitation wavelength of 350 nm. Hereafter, due to the increase in excitation wavelength, fluorescent emission peak shifted to longer wavelength, along with gradual decrease of fluorescent intensity. As CDs are excitation dependent, their fluorescent activity could be supervised by both size and surface defects such as oxidation [59]. Also, the discussed findings have been composed of previous studies [52,58,59].

CDs quantum yield assessment has reported about 43%. Self-surface passivation of CDs with distinctive elements leads to such great QY. This property could potentially promote CDs’ optical attribution. Elements that modify CDs play an important role in CDs’ functionalization [66]. It has been claimed in various reports that CDs synthesized from natural and green precursors have a parallel (1% to 50%) range of QYs [53]. Hence, our synthesized carbon dots could be counted in this high QY boundary.

### Fluorescence quenching by different pesticides

In this study, CDs were applied as a nano-sensor with the purpose of detecting compounds used in agriculture. Despite the fact that there have been enormous researches implemented on CDs applications as industrial sensors, studies on using CDs as nano-sensors for agricultural pesticides are still insignificant.

**Fig 4:**
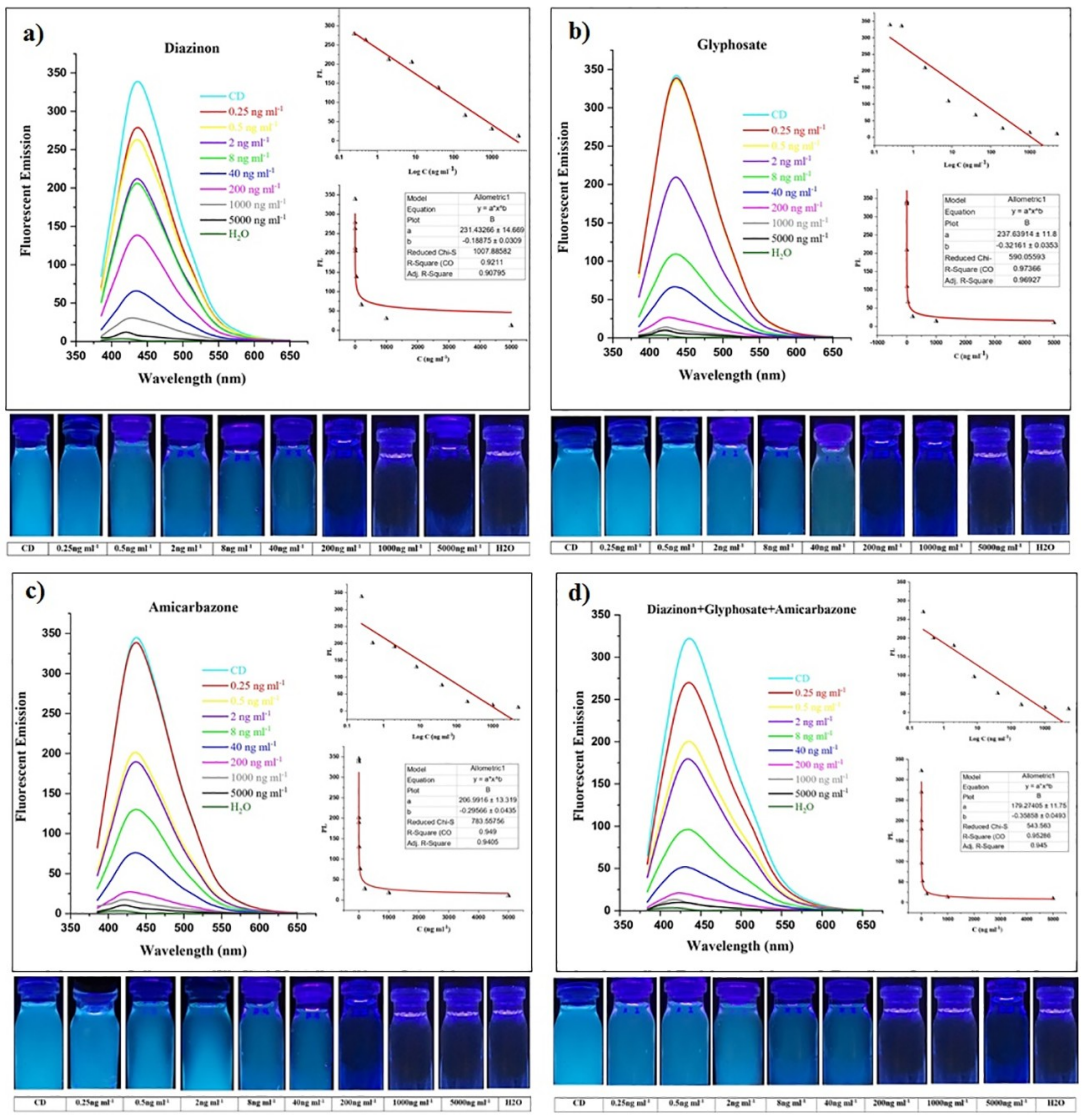
Fluorescent emission spectra of CDs in the presence of different concentrations of pesticides. Different concentrations of pesticides including 0.25, 0.5, 2, 8, 40, 200, 1000, and 5000 ng ml^-1^ in (A) diazinon, (B) glyphosate, (C) amicarbazone, and (D) all three pesticides at the same sample. Also, CDs have been selected as positive control owing to highest fluorescent emission intensity and ultrapure water was considered as negative control. Photographs present quenching intensity at different concentrations under the UV-light. The fluorescent emission and concentration of pesticides show sensitivity of these systems. Fluorescent emission was decreased due to increase in concentration of pesticides. The curves of fluorescence quenching between fluorescent emission and log C were analyzed.

In the past researches, gold (Au), and copper (Cu) NPs were used as nano-quencher to detect organophosphates. In comparison to previous nanomaterials, CDs are more available and more cost-effective. In addition to that, CDs show more accurate results in different real samples [9,67]. Herein, the fluorescence intensity of CDs was detected in the presence of different concentrations of pesticides. The interaction between CDs and pesticides result in fluorescence quenching which is dependent to pesticides concentration including 0.25, 0.5, 2, 8, 40, 200, 1000, 5000 ng ml^-1^. Therefore, pesticides concentration can be recognized by quenching intensity. A significant decrease in fluorescence intensity of CDs could be observed with naked eyes under a UV lamp. As shown in Fig 4, the addition of diazinon, glyphosate, and amicarbazone into CDs solution has caused fluorescent intensity to quench with a concentration-dependent manner. According to Fig 4A, the fluorescent intensity of CDs-diazinon has significantly decreased with an increase in diazinon concentration from 0.25 to 5000 ng ml^-1^. Meanwhile, the solution changes from bright blue to colorless under a UV lamp by having an increase in diazinon concentration which can be seen with the naked eye. Glyphosate quenching efficiency was not as much as diazinon. Fig 4B shows that 0.25 and 0.5 ng ml^-1^ of this compound have not any significant impact on the fluorescent quenching, while with an increase in concentration from 2 to 5000 ng ml^-1^, emission peaks decreased significantly. The results were also observed under UV- light. Fig 4C shows a significant decrease in fluorescent intensity at emission peaks with an increasing concentration range of amicarbazone (0.5–5000 ng ml^-1^). When excitation was performed at 365 nm, the fluorescence changes could be observed by the naked eye as well. Compared with CDs fluorescent intensity, 0.25 ng ml^-1^ of amicarbazone has not made any significant difference in fluorescent emission. As it was expected, high concentration leads to more fluorescent quenching.

The three mentioned pesticides were examined at the same sample (Fig 4D). At the lowest concentration (0.25 ng ml^-1^) the fluorescence intensity is perceptible. By increasing the concentration, CDs would be fully quenched that presents the resemblance when the test was carried out with diazinon. However, three pesticides at same sample could be detected with lower concentration comparing to amicarbazone and glyphosate with 0.5 and 2 ng ml^-1^ of quenching start-point in respect. Moreover, tested pesticides showed no reduction effect on each other’s fluorescent intensity when all of three pesticides were presented at the same sample.

**Fig 5:**
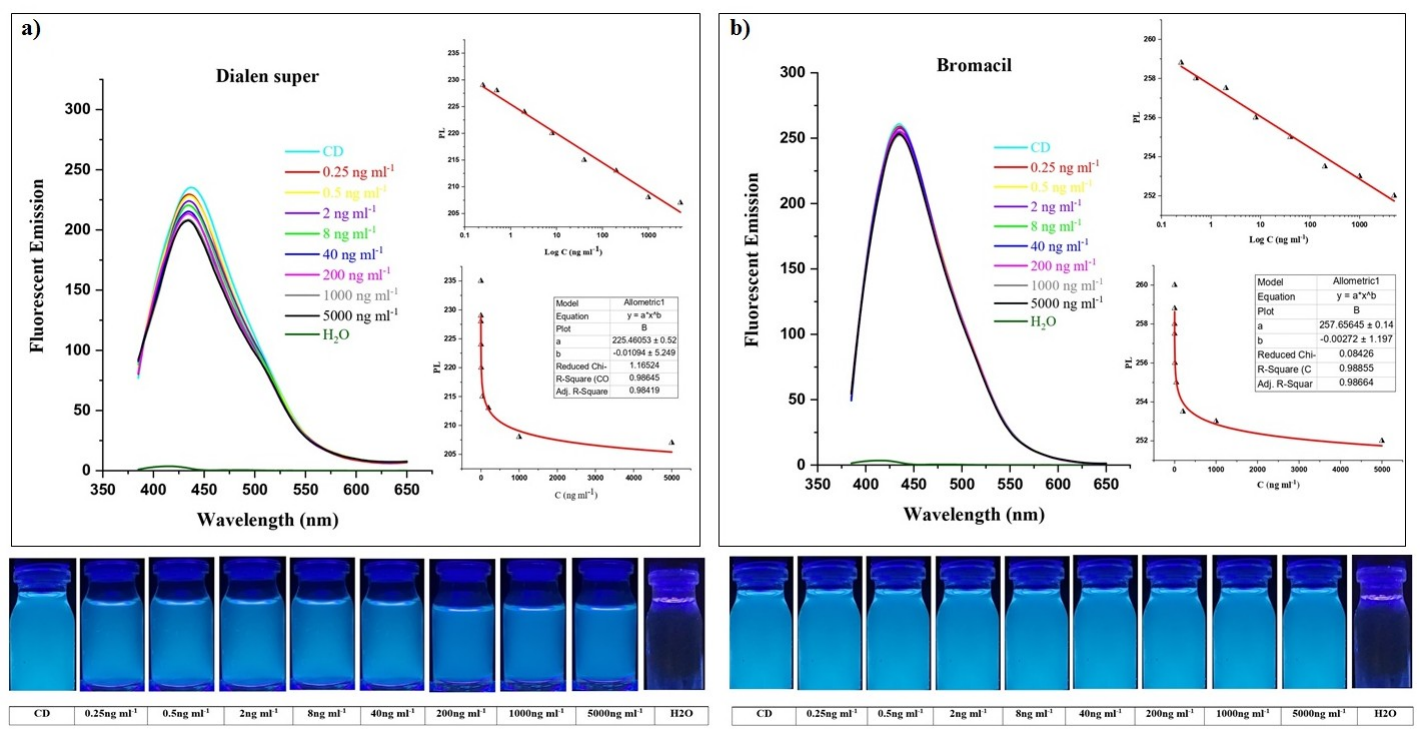
Fluorescent emission spectra of CDs in the presence of dialen super and bromacil pesticides. Different concentrations of pesticides including 0.25, 0.5, 2, 8, 40, 200, 1000, and 5000 ng ml^-1^ in (A) dialen super and (B) beolacil. Two pesticides showed no significant reduction on fluorescent intensity under UV light. The curves of fluorescence quenching between fluorescent emission and log C were analyzed.

Furthermore, the specificity was examined by monitoring the change of fluorescence emission intensity of CDs in the presence of dialen super and bromacil pesticides. It has been recorded that dialen super pesticide did not significantly affect the CDs fluorescence intensity (Fig 5a). By increasing the concentration, the quenching intensity showed trivial change which is negligible compared to amicarbazone, glyphosate, and diazinon quenching potential. In addition to that, increase in dialen super presented a minor fluorescence quenching which could be considered as insignificant when it is compared to other three pesticides. The graph shows the insignificancy of the bromacil in CDs fluorescence quenching. By increasing the concentration from 0.25 to 5000 ng ml^-1^, no significant change was observed (Fig 5b). As mentioned above, quenching effect of these substances was negligible which confirms specificity of CDs to detect glyphosate, diazinon and amicarbazone. For detecting the potential of sensitivity from peaks of each graph, the curve fitting plots were analyzed. Fluorescent emission of CDs versus concentration of pesticides (C) were plotted with power nonlinear curves that highlight the quenching level. Also, the range of each “adj R square” has been reported. Furthermore, the interaction between fluorescent emission and log concentration of pesticides with linear behavior has been displayed in Figs 4 and 5 (by y = a+b^x^ equitation).

Compared with other pesticide detection methods based on fluorescence characteristics of quantum dot materials, the limit of detection of this report was lowest. Also, this method was sensitive and had a wide detection range [64]. To the best of our knowledge, the detection limits that have been observed in other reports were 20–280 μg L^-1^. Therefore, it could be concluded that green synthesized CDs exhibit more promising biocompatibility in contrast to CDs derived from other precursors [2,10,18,68,69]. Furthermore, analytical performance of the proposed method has been compared with some typical sensing methods for mentioned pesticides in Table 1.

**Table 1:** Analytical features of some typical sensing methods employed for glyphosate, diazinon, and amicarbazone determination.

Pesticides	Analytical Methods	Detection limit	References
**Glyphosate**	High performance chromatography (HPLC)	50 ng ml^-1^	[70]
	Gas chromatography- mass spectrometry (GC-MS)	0.1 μg ml^-1^	[71]
	capillary electrophoresis (CE)	85 ng ml^-1^	[72]
	Ion- chromatography	0.042 μg ml^-1^	[73]
**Diazinon**	High performance chromatography (HPLC)	0.5 ng ml^-1^	[74]
	Liquid chromatography-UV (LC/UV)	2.96 ng ml^-1^	[75]
**Amicarbazone**	Liquid Chromatography/Mass Spectrometry (LC-MS/MS)	5 μg kg^-1^	[76]

### Pesticide and CDs interaction in real sample

**Fig 6:**
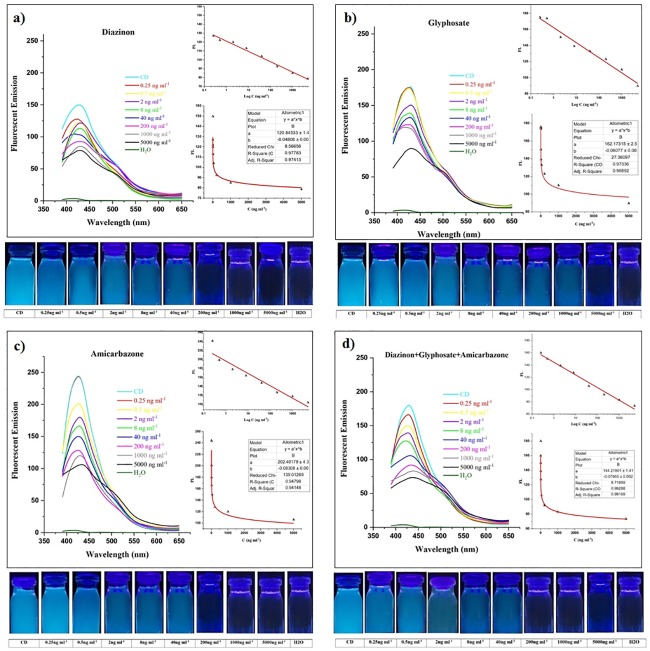
Fluorescence intensity spectra of CDs at various concentrations of pesticides. Different concentrations of pesticides including 0.25, 0.5, 2, 8, 40, 200, 1000, and 5000 ng ml^-1^ in real sample (cherry tomato juice) depicted by using origin software (A) diazinon, (B) glyphosate, (C) amicarbazone, and (D) all three pesticides at the same sample. cherry tomato juice was considered as negative control and pesticide free CDs-tomato juice solution was selected as positive control. The interaction between fluorescent emission and log C was observed. The images show quenching intensity increment by the increase in pesticides concentration which is observable under UV light. Fluorescent emission versus concentration of pesticides (C) were plotted with power nonlinear curve to explain sensitivity.

To evaluate the feasibility of this method, the fluorescent sensing behavior of CDs for diazinon, glyphosate, and amicarbazone in real fruit samples was investigated. Herein, pesticide-free cherry tomatoes were exposed to different concentrations of mentioned pesticides and the extracted tomatoes juice were observed under 365nm UV light after 5 days. Fig 6 explains that diazinon, glyphosate, and amicarbazone exhibit quenching behavior at the presence of carbon dots in tomato juice. Also, the solution containing only CDs and cherry tomato juice was considered as positive control and the cherry tomato juice was selected as negative control. However, the quenching intensities at cherry tomato juice were lower than the solution that only contains CDs and pesticides. This is due to the fact that with the penetration of CDs into tomatoes, the concentration of free CDs in the solution would be diluted. Therefore, fluorescence intensity would be slightly reduced. It could be observed in Fig 6A that by increasing of diazinon concentration in CDs-cherry tomatoes solution, fluorescence emission intensity has been significantly decreased. This also could be confirmed by images that have been taken under UV-light. Raising up the concentrations of diazinon from 0.25 ng ml^-1^ to 5000 ng ml^-1^ caused the blue color to change into colorless under UV-light. On the other hand, glyphosate fluorescence quenching effect has been observed in more concentration ranges than diazinon. With 0.25 ng ml^-1^ and 0.5 ng ml^-1^ of glyphosate, no significant change in CDs fluorescence emission was observed. However, the fluorescence quenching gradually started when the concentration of glyphosate reached to 2 ng ml^-1^ and by increasing the concentration, promotion in the fluorescence quenching has been observed. Also, images taken under UV-light verify the color change in high dosages of glyphosate (Fig 6B). It could be observed in Fig 6C that the fluorescence quenching starts at 0.5ng ml^-1^ of amicarbazone and no significant quenching could be found in 0.25 ng ml^-1^. Moreover, images approve the process of downgrading fluorescence emission intensity with the increase of amicarbazone concentration. As expected, test carried out with the presence of three pesticides in one sample shows similarity in result of diazinon which presented fluorescence quenching at the lowest concentration. Comparing to amicarbazone and glyphosate with the 0.5 and 2 ng of quenching start point, the quenching initiation of the three pesticides in one sample could be recorded in lower concentration (Fig 6D).

Non-linear curve fitting has been analyzed for these systems and their sensitivity has been illustrated (Fig 6). Moreover, linear behavior of each system was plotted by fitting to the y = a+b^x^ equitation. The above discussed reports have divulged that this detection method is accurate and repeatable for the determination of these pesticides. Additionally, the results are harmonious with previous reports [2,16,77].

## Conclusion

As the pesticide residues have caused a global concern over mankind’s health and the environment, an accurate and expeditious detection method should be at the top priority. Carbon dots derived from cauliflower precursor and hydrothermal synthesis technique were synthesized as a nano-sensor to detect pesticides. The most encouraging advantages of CDs-based fluorescent sensors are that they are simple to form, non-toxic, time-saving, and inexpensive for industrial uses. The synthesized carbon dots have the potential to quench fluorescence emission intensity considerably in the presence of pesticides. The limit of detection is estimated to be 0.25, 0.5, and 2 ng ml^-1^ for diazinon, amicarbazone, and glyphosate respectively, which is considered the lowest limit of detection among typical detection method such as different kinds of chromatography, capillary electrophoresis, and carbon dots detection in previous studies. The selectivity of the prepared sensor could be confirmed by the absence of CDs fluorescence quenching, when they interfere with dialen super and bromacil. The synthesized CDs have the ability to detect glyphosate, amicarbazone and diazinon. Moreover, the presence of three pesticides in a one sample has also exhibits increase in quenching. However, it is possible that CDs may not be able to distinguish the pesticides in a sample containing the three pesticides. In addition to that, this method could be applied for the detection of these compounds in fruits and other agricultural products. Moreover, it is expected that by the worldwide use of such nano-sensor, many infected consumable products could be detected and human health would gain promotion.
